# Is Fluoxetine Good for Subacute Stroke? A Meta-Analysis Evidenced From Randomized Controlled Trials

**DOI:** 10.3389/fneur.2021.633781

**Published:** 2021-03-22

**Authors:** Guangjie Liu, Xingyu Yang, Tao Xue, Shujun Chen, Xin Wu, Zeya Yan, Zilan Wang, Da Wu, Zhouqing Chen, Zhong Wang

**Affiliations:** ^1^Department of Neurosurgery & Brain and Nerve Research Laboratory, The First Affiliated Hospital of Soochow University, Suzhou, China; ^2^Department of Neurology, The First Affiliated Hospital of Soochow University, Suzhou, China; ^3^Department of Neurosurgery, Yixing People's Hospital, Yixing, China

**Keywords:** stroke, recovery, rehabilitation, meta-analysis, fluoxetine, MRS

## Abstract

**Background and Purpose:** Fluoxetine is a drug commonly used to treat mental disorders, such as depression and obsessive–compulsive disorder, and some studies have shown that fluoxetine can improve motor and function recovery after stroke. Therefore, we performed a meta-analysis to investigate the efficacy and safety of fluoxetine in the treatment of post-stroke neurological recovery.

**Methods:** PubMed, Embase, and Cochrane Library were searched for randomized controlled trials (RCTs) that were performed to assess the efficacy and safety of fluoxetine for functional and motor recovery in subacute stroke patients up to October 2020. Review Manager 5.3 software was used to assess the data. The risk ratio (RR) and standardized mean difference (SMD) were analyzed and calculated with a fixed effects model.

**Results:** We pooled 6,788 patients from nine RCTs. The primary endpoint was modified Rankin Scale (mRS). Fluoxetine did not change the proportion of mRS ≤ 2 (*P* = 0.47). The secondary endpoints were Fugl-Meyer Motor Scale (FMMS), Barthel Index (BI), and National Institutes of Health Stroke Scale (NIHSS). Fluoxetine improved the FMMS (*P* < 0.00001) and BI(*P* < 0.0001) and showed a tendency of improving NIHSS (*P* = 0.08). In addition, we found that fluoxetine reduced the rate of new-onset depression (*P* < 0.0001) and new antidepressants (*P* < 0.0001).

**Conclusion:** In post-stroke treatment, fluoxetine did not improve participants' mRS and NIHSS but improved FMMS and BI. This difference could result from heterogeneities between the trials: different treatment duration, clinical scales sensitivity, patient age, delay of inclusion, and severity of the deficit.

## Introduction

Stroke is still a major cause of mortality and disability worldwide, leading to substantial burden of economic costs for treatment and post-stroke care (1). There is a huge research space for treatment to improve the function and motor recovery of stroke patients and reduce the disability rate of patients (2). The selective serotonin reuptake inhibitors (SSRIs) are highly regarded.

In addition to the widely used effect of antidepression, SSRIs might improve the function recovery through a range of mechanisms, including the stimulation of neurogenesis, anti-inflammatory neuroprotection, improving cerebral blood flow, and regulating the adrenergic system (3). Apart from the research based on the animal models, there is also clinical evidence of the effect for post-stoke function recovery. The SSRIs appeared to reduce disability scores regardless of risk of bias in a meta-analysis of 63 trials published by the Cochrane collaboration (4). Fluoxetine, sertraline, paroxetine, and citalopram were included in this analysis, among which fluoxetine was the most studied.

A large number of trials investigating the effects of fluoxetine on stroke and post-stroke are underway or have been completed. Early in 2011, (5) published the result of a double-blind, placebo-controlled trial testing whether fluoxetine enhanced motor recovery (FLAME trial). The outcome of 118 patients after 3 months of intervention showed a significant improvement of Fugl-Meyer Motor Scale (FMMS) in the fluoxetine treatment group compared to that in the placebo group (6). Later, several larger randomized controlled trials (RCTs) were launched to investigate more about the effects. Dennis et al. designed the FOCUS trial in 103 hospitals in the UK, including 3,127 patients treated for 6 months. They chose modified Rankin Scale (mRS) as the primary outcome. It came out that the distribution across mRS at 6 months was similar in the fluoxetine and placebo groups (7). Recently, two large trials, the AFFINITY trial (8) and EFFECTS trial (9), had published their results and also reported similar distribution of the mRS categories. There is also a meta-analysis published in 2019 (10), yet the two large trials with another recruited trial named FMRICH (11) had not been finished. Now, the data of FMRICH trial are also available (12).

Whether to use fluoxetine routinely in post-stroke treatment remained controversial. We did this meta-analysis based on the clinical trials mentioned above to provide more precise estimates of the efficacy as well as the safety of fluoxetine for stroke recovery.

## Methods

### Study Protocol

Before the project started, we drafted a research protocol following the Cochrane Collaboration format (13).

### Eligibility Criteria

We set the inclusion criteria as follows: (1) study type: RCT; (2) language restriction: only available in English; (3) participants: patients were eligible if they were aged 18 years or older with a clinical diagnosis of ischemic or hemorrhagic stroke in the previous 2–15 days; (4) intervention: 20 mg fluoxetine taken orally daily for 3–6 months and the corresponding control (placebo); (5) outcomes: efficacy outcomes including the mRS, the National Institutes of Health Stroke Scale (NIHSS), FMMS, and the Barthel Index (BI); safety outcomes including adverse events (AEs) and serious adverse events (SAEs). Included RCTs were not requested to supply all the outcomes mentioned above.

We set the exclusion criteria as follows: (1) study type: retrospective studies, cohort studies, case reviews, and case reports; (2) participants: patients with serious complications (such as depression); (3) control: active control (i.e., that a known, effective treatment as opposed to a placebo is compared to an experimental treatment).

### Search Strategy

Two independent investigators (GJL and XYY) systematically searched three main databases including MEDLINE, Embase, and Cochrane Library to identify relevant studies published until October 2020. The following search strategy was used: (fluoxetine[Title/Abstract]) AND (stroke[Title/Abstract]) for MEDLINE; “fluoxetine”/exp AND “stroke”/exp for Embase; “fluoxetine” in Title Abstract Keyword AND “stroke” in Title Abstract Keyword for Cochrane Library. Additionally, the reference lists of RCTs, relevant systematic reviews, and meta-analyses were also screened independently and manually to ensure a more comprehensive search.

### Study Selection and Data Collection

According to the eligibility criteria listed above, two reviewers (GJL and XYY) independently evaluated all study records from the three electronic databases and the reference lists of RCTs and relevant systematic reviews or meta-analyses. The duplicates and the research articles that only provided abstracts were excluded. A third reviewer (TX) who did not participate in the process of data collection would make the final decision of the disputed data when disagreements emerged among the two reviewers. After meticulous selection and evaluation, all data from the included RCTs were extracted as follows: basic information and outcome events included for each trial (Table 1), all inclusion and exclusion criteria, efficacy and safety outcomes, conclusion, and data acquisition time were shown in the online supplementary materials (Supplementary Table 1).

**Table 1:** Characteristics of the included studies.

**Trials**	**Publication**	**Country/Center**	**No. of patients**	**Sex**	**Age [Mean (SD)]**	**Types of stroke**	**Dose and duration of fluoxetine**
Marquez-Romero et al. (12) (NCT01737541) FMRICH	Clinical neurology and neurosurgery	Three centers in Mexico	Fluoxetine (*n* = 14) Placebo (*n* = 16)	Female Fluoxetine 6 (20) Placebo 9 (30)	Fluoxetine 54 (7.4) Placebo 60.5 (13.3)	ICH	20 mg/day for 3 months
Lundström et al. (9) (NCT02683213) EFFECTS	The Lancet Neurology	35 units in Sweden	Fluoxetine (*n* = 750) Placebo (*n* = 750)	Fluoxetine Female 287 (38%) Male 463 (62%)Placebo Female 288 (38%)Male 462 (62%)	Fluoxetine 70.6 (11.3) Placebo 71.0 (10.5)	Fluoxetine Non-stroke 2 (<1%) IS 662 (88%) ICH 86 (12%) Placebo Non-stroke 1 (<1%) IS 650 (87%) ICH 99 (13%)	20 mg/day for 6 months
Hankey et al. (8) (ACTRN12611000774921) AFFINITY	The Lancet Neurology	43 units in Australia (*n* = 29), New Zealand (four), and Vietnam (10).	Fluoxetine (*n* = 642) Placebo (*n* = 638)	Fluoxetine Female 231 (36%)Male 411 (64%) Placebo Female 245 (38%)Male393 (62%)	Fluoxetine 63.5 (12.5) Placebo 64.6 (12.2)	Fluoxetine Non-stroke 3 (<1%) IS 549 (86%) ICH 90 (14%) Placebo Non-stroke 1 (<1%) IS 542 (85%) ICH 95 (15%)	20 mg/day for 6 months
Dennis et al. 2019 (ISRCTN83290762) FOCUS	The Lancet	103 hospitals in the UK	Fluoxetine (*n* = 1,564) Placebo (*n* = 1,563)	Fluoxetine Female 589 (38%) Male 975 (62%) Placebo Female 616 (39%) Male 947 (61%)	Mean Fluoxetine 71.2 (12.4) Placebo 71.5 (12.1)	Fluoxetine Non-stroke 2 (0%) IS 1,410 (90%) ICH 154 (10%) Placebo Non-stroke 2 (0%) IS 1,406 (85%) ICH 157 (15%)	20 mg/day for 6 months
Chollet et al. (5) (NCT00657163) FLAME	The Lancet Neurology	Nine stroke centers in France	Fluoxetine (*n* = 59) Placebo (*n* = 59)	Male Fluoxetine 37 (63%) Placebo 35 (59%)	Fluoxetine 66.4 (11.7) Placebo 62.9 (13.4)	IS within the past 5–10 days	20 mg/day for 3 months
Bonin et al. (14) (NCT02208466)	Neurorehabilitation and neural repair	One center in the USA	Fluoxetine (*n* = 10) Placebo (*n* = 8)	Female Fluoxetine 5 (50%)Placebo 2 (25%)	Fluoxetine 50.5 (16.57) Placebo 57.38 (9.96)	IS	20 mg/day for 90 days
Asadollahi et al. (15) (IRCT20141116019971N3)	Clinical rehabilitation	A university-affiliated teaching hospital in Tehran, Iran	Fluoxetine (*n* = 30) Placebo (*n* = 30)	Fluoxetine Male 15 (50)Female 15 (50)Placebo Male 18 (60)Female 12 (40)	Fluoxetine 60.2 (8.52) Placebo 61.7 (9.6)	A first-time acute IS within the past 24 h	20 mg/day for 90 days
He et al. (16) (ChiCTR-TRC-12002078)	Journal of Stroke and Cerebrovascular Diseases	China	Fluoxetine (*n* = 187) Placebo (*n* = 187)	Male Fluoxetine 129 (72.1%)Placebo 120 (70.2%)	Fluoxetine 60.46 (10.35) Placebo 62.66 (11.69)	IS	20 mg/day for 90 days
Mikami et al. (17)	American Journal of Geriatric Psychiatry	The USA	Fluoxetine (*n* = 21) Nortriptyline (*n* = 15) Placebo (*n* = 26)	Male Treatment 34 (63.0) Placebo 17 (58.6)	Treatment 65.7 (12.4) Placebo 72.5 (9.4)	56 depressed and 48 non-depressed enrollees after stroke in the previous 6 months	10 mg/day for the first 3 weeks, 20 mg/day for weeks 4–6, 30 mg/day for weeks 7–9, and 40 mg/day for the final 3 weeks
Guo et al. (18) (ChiCTR-IPR-15007658)	Restorative Neurology and Neuroscience	China	Group A (*N* = 92) Group B (*N* = 85) Group C (*N* = 90)	Male Group A 67 (72.8%) Group B 61 (71.8%) Group C 66 (73.3%)	Group A 59.52 (10.52) Group B 61.51 (10.25) Group C 60.51 (11.69)	IS (First onset of stroke within 1 week)	Group A received fluoxetine 20 mg/day immediately; Group B received fluoxetine 20 mg/day 7 days after enrollment; and group C did not receive fluoxetine.
Pariente et al. (19)	Annals of Neurology	France	Placebo-controlled crossover 8	Three women and five men	Mean age, 61.7 years; range, 43–75 years	All patients had a single ischemic lacunar infarction assessed by computed tomography (CT) scan	Single 20 mg dose
Robinson et al. (20)	American Journal of Psychiatry	USA and Argentina	Non-depressed Fluoxetine (*N* = 17) Placebo (*N* = 16)	Non-depressed Female Fluoxetine 2 (12%) Placebo 4 (25%)	Non-depressed Fluoxetine 66 (13) Placebo 67 (9)	All pathological types, within 6 months	Dose increased over 3 weeks from 10 to 30 mg daily; total 12 weeks
Dam et al., (21)	Stroke; a journal of cerebral circulation	Italy	Fluoxetine (*N* = 16) Placebo (*N* = 16)	M/F Fluoxetine 7/9 Placebo 7/9	Fluoxetine 67.5 (8.9) Placebo 68.4 (5.5)	Ischemic stroke, 1–6 months	20 mg daily for 12 weeks

*FMRICH, Fluoxetine for Motor Recovery after acute Intracerebral Hemorrhage; EFFECTS, the Efficacy oF Fluoxetine—a randomized Controlled Trial in Stroke; AFFINITY, the Assessment oF FluoxetINe In sTroke recoverY; FOCUS, Fluoxetine Or Control Under Supervision; FLAME, FLuoxetine for motor recovery After acute ischaeMic strokE; ICH, intracerebral hemorrhage; IS, ischemic stroke*.

### Risk of Bias

The risk of bias plot for individual studies was assessed with the Review Manager 5.3 software. The uniform criteria to assess the risk of bias for RCTs of the Cochrane Collaboration were applied, which included selection bias, performance bias, detection bias, attrition bias, reporting bias, and other potential biases. Each bias criterion was classified as “low,” “high,” or “unclear” after independently judged by the third reviewer.

### Summary Measures and Synthesis of Results

We use Review Manager 5.3 for data analysis. Statistical heterogeneity was estimated by I^2^ statistic. All analyses used a fixed effects model. Risk ratios (RRs) were used for dichotomous variables, and standardized mean differences (SMDs) were used for continuous variables. P < 0.05 was considered statistically significant. Heterogeneity was estimated *via* the I^2^ statistic, which was as follows: I^2^ <30% suggests “low heterogeneity”; I^2^ between 30 and 50% means “moderate heterogeneity”; I^2^ > 50% denotes “substantial heterogeneity.” Sensitivity analysis was used to explore the stability of the consolidated results. For all the analyses, two-tailed tests were performed and a *P*-value < 0.05 was considered statistically significant.

### Outcome of Interest

Primary efficacy outcome was the disability assessed by the mRS at the end of the treatment. The higher scores represented more severe disability. We compared the proportion of patients with better function recovery (mRS 0–2). Secondary efficacy outcomes included motor recovery assessed by FMMS and activities of daily living assessed by BI and NIHSS. We focused on the change of these scores. For safety outcomes, we chose some AEs and SAEs that were often reported by clinical trials, such as new-onset depression, new antidepressants, fractures, hyponatremia, seizure, death, suicide, any stroke, fall with injury, any bleeding events, any thrombotic events, hyperglycemia, hypoglycemia, nausea, insomnia, and diarrhea.

## Results

**Figure 1:**
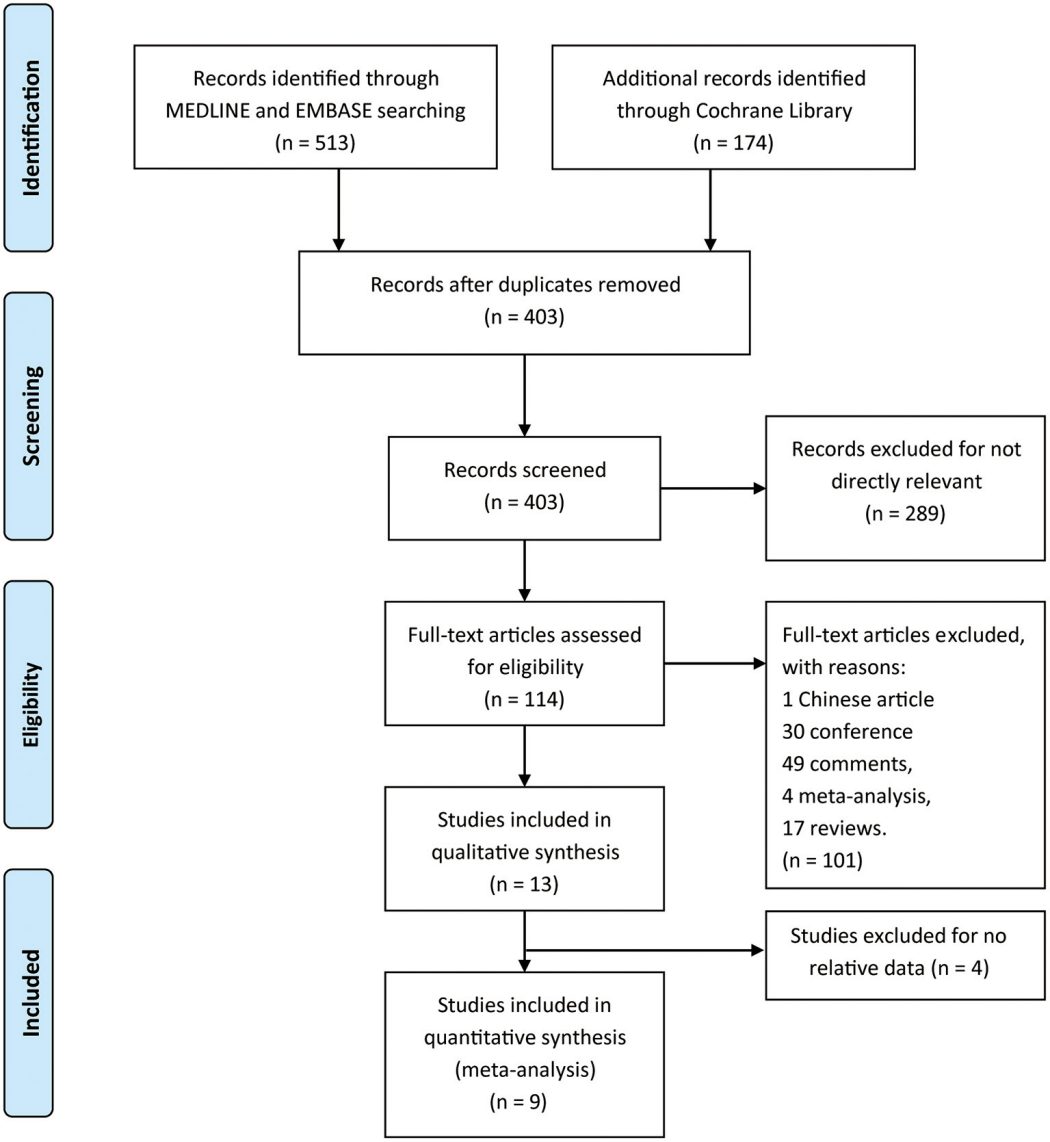
The study search, selection, and inclusion process.

We identified 687 references from the database searches. However, 284 duplicates and 289 irrelevant records were removed. Here, 114 articles were assessed through full text for eligibility, among which one article in Chinese, 30 conference abstracts, 49 comments, four meta-analyses, and 17 reviews were excluded. Thirteen articles were included in the analysis, and four were finally excluded because the data could not be integrated, but all were referenced (Figure 1). The eligible trials included five multicenter and four single-center trials. The baseline characteristics for each study were listed in Table 1.

### Efficacy Outcomes

**Figure 2:**
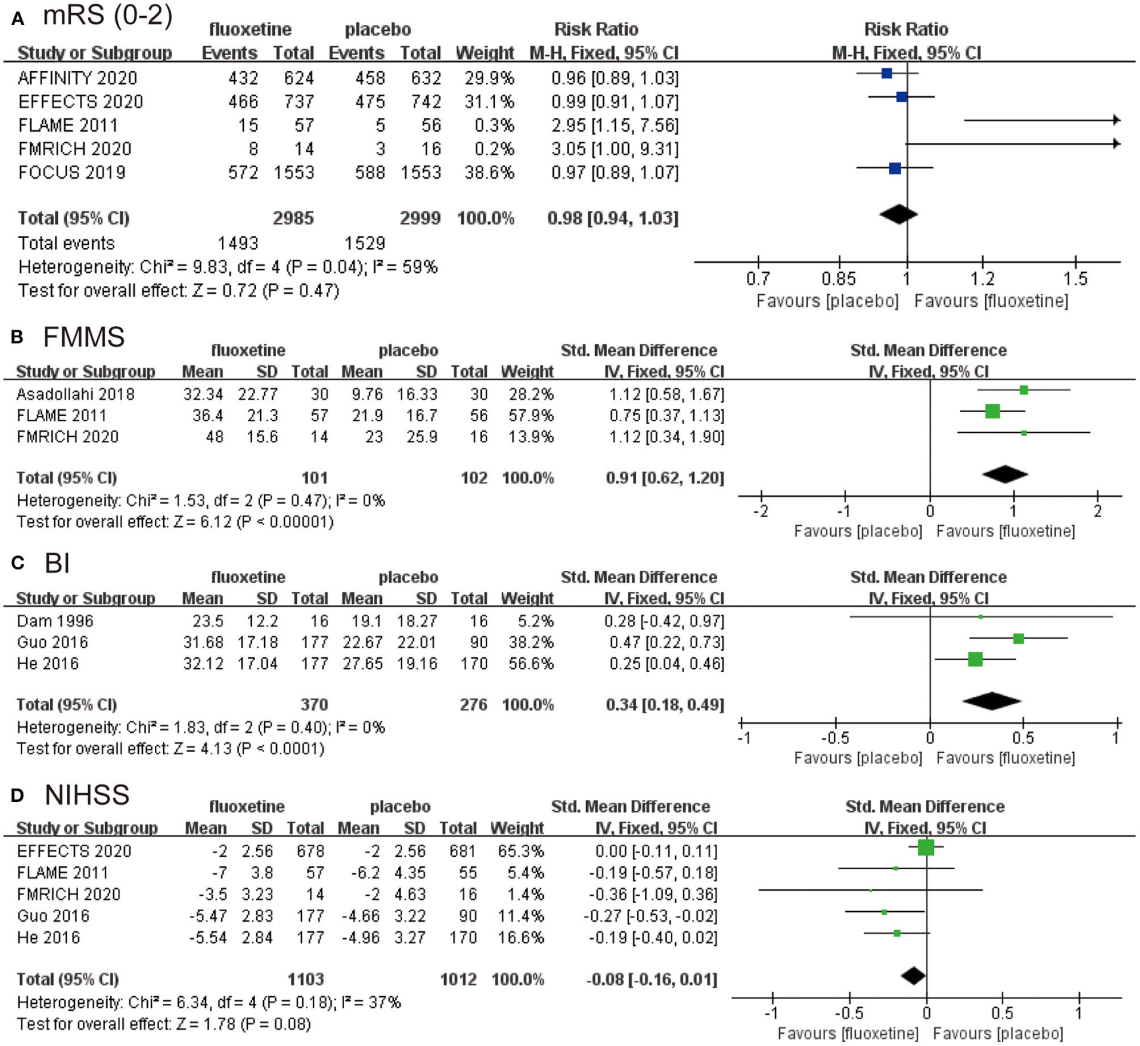
The pooled RR or SMD of primary outcomes and secondary outcomes. The blue square indicates the estimated RR. The green square indicates the estimated SMD. The size of blue square indicates the estimated weight of each RCT, and the extending lines indicate the estimated 95% CI of RR for each RCT. The black diamond indicates the estimated RR (95% CI) for all patients together. **(A)** Modified Rankin Scale (mRS; 0–2). **(B)** Fugl-Meyer Motor Scale (FMMS). **(C)** Barthel Index (BI). **(D)** National Institutes of Health Stroke Scale (NIHSS). RR, risk ratio; SMD, standardized mean difference; RCT, randomized controlled trial; CI, confidence interval.

We combined data for the outcome of independence on mRS 0–2 using an RR with a fixed effects model (RR 0.98, 95% CI 0.94–1.03, *P* = 0.47; five studies, 5,984 participants, I^2^ = 59%). The result demonstrated no difference in independence on mRS between fluoxetine treatment and placebo (Figure 2A).

We calculated the change of FMMS from baseline to end, and the data were then combined (SMD 0.91, 95% CI 0.62–1.20, *P* < 0.00001; three studies, 203 participants, I^2^ = 0), which was in favor of fluoxetine group (Figure 2B). The change of BI assessing activities of daily living also supports the efficacy of fluoxetine (SMD 0.34, 95% CI 0.18–0.49, *P* < 0.0001; three studies, 646 participants, I^2^ = 0) (Figure 2C), as well as the NIHSS (SMD −0.08, 95% CI −0.16–0.01, *P* = 0.08; five studies, 2,115 participants, I^2^ = 37%) (Figure 2D). The data from FMRICH are means and SDs estimated from reported medians and interquartile ranges. The trial of He (2016) carries a high risk of bias. We recalculated the results for BI and NIHSS without data from He (2016) and got similar outcomes. The results were provided in the supplementary materials (Supplementary Figures 2, 3).

### Safety Outcomes

We combined the data of reported AEs from all trials using fixed effects models. The results are shown in Table 2.

**Table 2:** Effect sizes from meta-analysis of safety outcomes; from all trials using fixed effects models.

**Safety outcomes**	**Number of trials (number of participants) contributing to the meta-analysis**	**RR [95% CI]**	***P*-value**	**I^**2**^ (%)**
Death	4 trials (*n* = 6,257)	1.10 [0.71, 1.70]	0.88	0
Suicide	3 trials (*n* = 5,907)	0.82 [0.24, 2.83]	0.75	0
Any stroke	3 trials (*n* = 5,907)	0.93 [0.72, 1.20]	0.58	30
Bone fracture	3 trials (*n* = 5,907)	2.30 [1.59, 3.32]	<0.0001	0
Fall with injury	2 trials (*n* = 4,407)	1.71 [0.80, 3.64]	0.16	68
New depression	3 trials (*n* = 5,907)	0.75 [0.65, 0.86]	<0.0001	0
New antidepressant	3 trials (*n* = 5,907)	0.77 [0.67, 0.87]	<0.0001	0
Seizure	5 trials (*n* = 6,370)	1.44 [1.05, 1.97]	0.03	19
Any bleeding events	4 trials (*n* = 6,257)	1.22 [0.85, 1.73]	0.28	0
Any thrombotic events	3 trials (*n* = 5,907)	0.84 [0.67, 1.05]	0.13	23
Hyponatremia	4 trials (*n* = 6,020)	2.00 [1.15, 3.45]	0.01	21
Hyperglycemia	3 trials (*n* = 5,907)	0.90 [0.54, 1.50]	0.69	83
Hypoglycemia	2 trials (*n* = 4,407)	1.77 [0.90, 3.48]	0.10	0
Nausea	2 trials (*n* = 143)	5.28 [0.92, 30.15]	0.06	0
Insomnia	4 trials (*n* = 542)	1.13 [0.83, 1.55]	0.44	0
Diarrhea	3 trials (*n* = 493)	1.17 [0.60, 2.27]	0.64	0

*RR, risk ratio; CI, confidence interval*.

Fluoxetine reduced new-onset depression (RR 0.75, 95% CI 0.65–0.86, *P* < 0.0001; three trials, 5,907 participants, I^2^ = 0) and use of new antidepressant (RR 0.77, 95% CI 0.67–0.87, *P* < 0.0001; three trials, 5,907 participants, I^2^ = 0) in patients, which was associated with the antidepressant effect of SSRIs.

However, compared with the placebo group, fluoxetine treatment group was at higher risk of bone fracture (RR 2.30, 95% CI 1.59–3.32, P < 0.0001; three trials, 5,907 participants, I^2^ = 0). Another adverse effect with significant difference was hyponatremia (RR 2.00, 95% CI 1.15–3.45, *P* = 0.01; four trials, 6,020 participants, I^2^ = 21%). The standard of hyponatremia was <130 mmol/L in the EFFECTS trial, <125 mmol/L in the AFFINITY and FOCUS trials, and unreported in the FLAME trial. The fluoxetine group also had more seizure events (RR 1.44, 95% CI 1.05–1.97, *P* = 0.03; five trials, 6,370 participants, I^2^ = 19%).

The EFFECTS trial reported more falls causing injury in the fluoxetine group, but the data combined with the AFFINITY trial did not show any difference.

There was no significant difference between fluoxetine and placebo groups in severe adverse effects, such as death (RR 1.10, 95% CI 0.71–1.70, *P* = 0.88; four trials, 6,257 participants), suicide (RR 0.82, 95% CI 0.24–2.83, *P* = 0.75; three trials, 5,907 participants), or stroke (RR 0.93, 95% CI 0.72–1.20, *P* = 0.58; three trials, 5,907 participants, I^2^ = 30%). In addition, fluoxetine did not increase or decrease the risk of bleeding events or thrombotic events. No significant difference was found in the glucose level and other adverse effects including nausea, insomnia, and diarrhea as well.

### Risk of Bias

**Figure 3:**
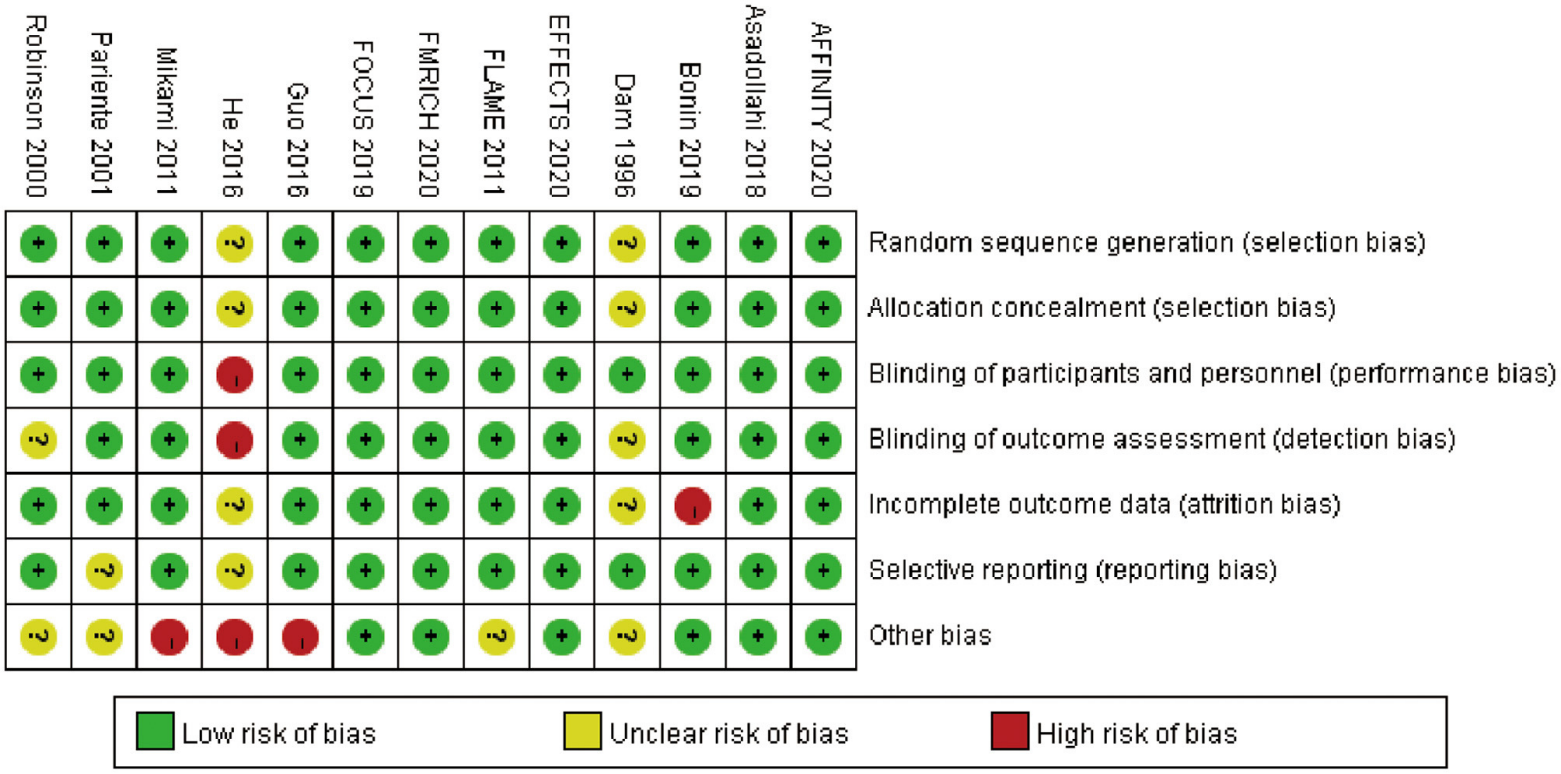
Risk of bias: a summary table for each risk of bias item for each study.

Full details of the risk bias for all enrolled studies were shown in Figure 3. Two clinical trials showed an unclear risk of bias both in random sequence generation and allocation concealment. For the blinding of participants and personnel, the risk of bias was high in one trial. For the blinding of outcome assessment, the risk of bias was high in one trial and unclear in two trials. For the incomplete outcome data, the risk of bias was high in one trial and unclear in two trials. For selective reporting, the risk of bias was unclear in two trials. For other biases, the risk was high in three trials and unclear in four trials.

## Discussion

Our meta-analysis of fluoxetine for stroke recovery identified nine RCTs recruiting 6,788 patients, and six (*n* = 6,115) were of high methodological quality. For efficacy outcomes, fluoxetine did not significantly improve patients' function recovery, but it might improve motor recovery. As for safety outcomes, it was found that fluoxetine increased the risk of fractures, seizure, and hyponatremia but reduced the risk of post-stroke depression compared to placebo.

Our primary efficacy outcome of mRS ≤ 2 showed no significant difference between fluoxetine and placebo groups. mRS is a scale used to assess disability in patients who suffered from stroke. It is divided into seven levels, with a lower score indicating better function recovery (22). mRS ≤ 2 was considered independence. The large trials used the proportion of mRS ≤ 2 as the primary outcome in common, which was adopted in our meta-analysis as well. Final result was negative, indicating that fluoxetine did not improve function recovery after stroke. Substantial heterogeneity was the result of three larger trials that were not the same as the other two. We also did a sensitivity analysis, which proved that the data were stable (Supplementary Figure 1). In addition, the trial of (17) reported improvement in mRS compared to placebo, but the data were illustrated in a line chart that could not be pooled in our meta-analysis.

Secondary efficacy outcomes included FMMS, BI, and NIHSS. Although the analysis of FMMS (*P* < 0.0001, *n* = 203, I^2^ = 0%; Figure 2B) and BI (*P* < 0.0001, *n* = 646, I^2^ = 0%; Figure 2C) provided positive outcomes with low heterogeneity, the results were based only on some RCTs of small scale, thus were less convincing. FMMS is an index designed to assess motor function, balance, sensation, and joint function in patients with post-stroke hemiplegia (23, 24). BI for daily activities of daily living is another assessment of disability or independence, with higher scores indicating better functional status (25). The change of FMMS and BI from baseline to the end of treatment described as ΔFMMS and ΔBI could reflect the degree of recovery.

Moreover, the *P*-value of NIHSS analysis was 0.08, which indicated that fluoxetine had a potential tendency on the improvement of NIHSS compared with placebo (Figure 2D). NIHSS is widely used to objectively rate the severity of stroke (26). Several RCTs of relatively small scale indicated a positive effect of fluoxetine, but in large RCTs, only EFFECTS provided NIHSS data, and the result was negative. It could be deduced that the result would become less significant with a larger sample size.

Previously, fluoxetine was mainly used to treat depression, obsessive–compulsive disorder, and other mental disorders. Several animal studies have found mechanisms by which fluoxetine has the potential to improve recovery of motor function (27). Firstly, SSRIs can increase neurogenesis and neurotrophin expression in the hippocampus to exert beneficial effects on the behavior of mice (28, 29). According to an animal study, SSRIs promote neurogenesis in the hippocampus and subventricular zone of the ventricular canal in mice where neurogenesis usually occurs (30). In addition, SSRI-mediated neurogenesis may contribute to structural and function recovery after cerebral ischemia and the migration of new neurons from the neurogenic zone to the injured zone (31, 32). Neurotrophins are proteins that promote organogenesis and embryogenesis and neuroplasticity (33). Secondly, SSRIs may have the ability to protect neurons by inhibiting inflammatory responses through inhibition of microglia and neutrophils (34, 35). Inflammation is the main cause of brain cell damage in the later stages of stroke (35). (36)When inflammation damages brain cells, SSRIs reduce the number of cytotoxic inflammatory molecules by decreasing the expression of microglia and neutrophils to protect brain cells from inflammatory damage. Animal experiments have shown that 9 h after stroke in mice using SSRIs, there is still a significant improvement in brain injury area volume and neurological function compared to the control group (37). This supports the idea that SSRIs improve mouse neurological function. Thirdly, SSRIs may improve the regulation of cerebral blood flow by increasing the expression of heme oxygenase-1 (HO-1) and hypoxia-inducible factor-1alpha (HIF-1alpha) (38). An animal study found that SSRIs increased HO-1 expression, which in turn led to the production of carbon monoxide, to regulate vascular tone independent of nitric oxide synthase-related pathways (39). Finally, animal studies have found an increase in β-1 adrenergic receptor expression in brain-injured regions of mice after administration of SSRIs (34), which may improve function recovery, but the mechanism of β-1 adrenergic upregulation in ischemic brain regions remains to be answered. From the results of our meta-analysis, the possible mechanisms of these animal experiments may not be applicable to humans (40). These trials did not test the effect of fluoxetine on neurogenesis, as regeneration of key nerve bundles may take months or years, and late endpoints were not assessed.

As for the safety outcomes, the results indicated that fluoxetine reduced the incidence of new depression and use of new antidepressants, which is closely related to its widely used effect in the treatment of depression. However, we also found that fluoxetine increased the risk of bone fracture, hyponatremia, and seizure in stroke patients.

Overall, according to our meta-analysis, the use of fluoxetine for recovery from stroke should be considered in view of the risk/benefit ratio. Therefore, we did not recommend prescribing fluoxetine for stroke patients at risk and/or who did not suffer from any mood disorders. However, for those patients who showed propensity of post-stroke depression, fluoxetine can be recommended to prevent post-stroke depression only when the AEs possibly caused by fluoxetine could be avoided.

Our meta-analysis included two up-to-date large RCTs: EFFECTS and AFFINITY. The total recruited patients were 6,788, which was nearly double of the most recent meta-analysis (10). Compared with previous relevant meta-analyses, the RCTs we included were published most recently and had a better experimental design. Among the RCTs we included, six trials were of high methodological quality, which reduced bias in all respects. FOCUS, EFFECTS, and AFFINITY all have more than a thousand participants. About four-fifths (*n* = 5,907) of the participants (*n* = 6,788) were from these three large trials. AFFINITY has multiethnic participants from 43 hospitals in three countries. It was designed to assess the effect of 6 months of daily oral fluoxetine on function recovery after stroke in an ethnically diverse population.

Our meta-analysis also has some limitations. Firstly, we included 13 RCTs that met the inclusion criteria, but due to inconsistencies in each trial testing participants' motor and function recovery, we ended up using data from only nine RCTs. The four RCTs, while not incorporating data processing for mapping, had little impact on the overall results and were referenced by us. When we worked with patient data, some mean values were estimated from the median value, which could affect the accuracy of the data. In included trials, there were variables that were not analyzed. The large trials also carried out some other health status assessments, such as Stroke Impact Scale and European Quality of Life questionnaire, but the results showed no significant difference between the experimental and control groups, thus they were not included in our analysis. The study performed by (21) used BI and another index named Hemispheric Stoke Scale Gait score to assess function and got positive outcomes, but only BI was involved in our meta-analysis. Some included studies also provide results for depression assessment, but we did not choose depression as an efficacy outcome, and these scores were not analyzed. Secondly, the heterogeneity was caused by several factors. The scores of each trial were not uniformly trained, and the investigators were not identical in their scoring criteria. The treatment duration of fluoxetine was 3 months in some trials and 6 months in others. In a pharmacological point of view, a 3-month treatment is not the same as a 6-month treatment, and outcomes may differ. This may affect the results on the mRS. Interestingly, all trials testing a 3-month treatment had positive effects on FMMS and BI with fluoxetine. Thirdly, meta-analyses were performed based on the number of patients in clinical trials and large trials. However, time-consuming clinical scales like the FMMS cannot be performed in large trials, although these scales are strongly recommended in recent guidelines (41). Less sensitive scales, such as the mRS are then used, but small effects may not be demonstrated. Our meta-analysis included both ischemic and hemorrhagic stroke participants, and in FLAME, all participants were ischemic stroke patients, but in EFFECTS, AFFINITY, and FOCUS, participants were both ischemic and hemorrhagic stroke patients. All participants in FMRICH were patients with hemorrhagic stroke. Different compositions of participants might have an inconsistent effect on the findings of FLAME and other experiments. In addition, a substantial proportion of patients recruited by three large trials (FOCUS, EFFECTS, AFFINITY) were not impaired enough. They might recover spontaneously, and no fluoxetine effect can be evidenced, as there will be a ceiling effect. These trials met our inclusion criteria and exhibited low heterogeneity, but *post hoc* analyses on very impaired patients are warranted from such large trials. Finally, we did not register the meta-analysis but used almost the same research methods as the previous meta. We performed advanced searches from PubMed, Embase, and Cochrane Library to have a complete search of the collated studies. We will update if new trials are published.

## Conclusion

In conclusion, the present study indicated that oral fluoxetine did not improve participants' mRS and NIHSS but improved FMMS and BI. Meanwhile, fluoxetine increased the risk of hyponatremia, fractures, and seizure but reduced the risk of new-onset depression. These conclusions could provide evidence for the application of fluoxetine in post-stroke treatment.

## Data Availability Statement

The original contributions generated for the study are included in the article/Supplementary Material, further inquiries can be directed to the corresponding authors.

## Author Contributions

ZhW and ZC were the principal investigators. GL and XY designed the study and developed the analysis plan. SC and XW analyzed the data and performed the meta-analysis. ZY and ZiW contributed in writing of the article. TX and DW revised the manuscript and polished the language. All authors read and approved the final submitted paper.

## Conflict of Interest

The authors declare that the research was conducted in the absence of any commercial or financial relationships that could be construed as a potential conflict of interest.

## Abbreviations

FMRICH: Fluoxetine for Motor Recovery after acute Intracerebral Hemorrhage
EFFECTS: the Efficacy oF Fluoxetine—a randomized Controlled Trial in Stroke
AFFINITY: the Assessment oF FluoxetINe In sTroke recovery
FOCUS: Fluoxetine Or Control Under Supervision
FLAME: FLuoxetine for motor recovery After acute ischaeMic stroke
ICH: intracerebral hemorrhage
IS: ischemic stroke
mRS: modified Rankin Scale
FMMS: Fugl-Meyer Motor Scale
NIHSS: National Institutes of Health Stroke Scale
BI: Barthel Index.
