# Supplementary material for: Is Fluoxetine Good for Subacute Stroke? A Meta-Analysis Evidenced From Randomized Controlled Trials
Source: Front Neurol. 2021 Mar 22;12:633781. doi: 10.3389/fneur.2021.633781 (PMC8019826; doi:10.3389/fneur.2021.633781)

Is Fluoxetine Good for Acute Stroke?: A Meta-Analysis Evidenced from Randomized Control Trial

Guangjie Liu, MM [1,#], Xingyu Yang, MM [1,#], Tao Xue, MM [1,#], Shujun Chen, MM [2], Xin Wu, MM [1], Zeya Yan, MM [1], Zilan Wang, MM [1], Da Wu, MM [3], Zhouqing Chen, MD, PhD [1,*], Zhong Wang, MD, PhD [1,*]

[1] *Department of Neurosurgery & Brain and Nerve Research Laboratory, The First Affiliated Hospital of Soochow University, Suzhou, Jiangsu Province, 215006, China*

[2] *Department of Neurology, The First Affiliated Hospital of Soochow University, Suzhou, Jiangsu Province, 215006, China*

[3] *Department of Neurosurgery, Yixing People's Hospital, Yixing 214200, China.*

Complete co-author email addresses:

Guangjie Liu, Email: 1937247527 @qq.com

Xingyu Yang, Email: yxystarby@163.com

Tao Xue, Email: 2992326676@qq.com

Shujun Chen, Email: 1026443927@qq.com

Xin Wu, Email: 15305130828@163.com

Zeya Yan, Email: Dr_Yzeya@163.com

Zilan Wang, Email: zlwang@stu.suda.edu.cn

Da Wu, Email: wudabrain@163.com

Zhouqing Chen, Email: zqchen6@163.com

Zhong Wang, Email: wangzhong761@163.com

[#]Guangjie Liu, Xingyu Yang and Tao Xue contribute equally to this work.

*Corresponding author: Zhouqing Chen or Zhong Wang Department of Neurosurgery, The First Affiliated Hospital of Soochow University, 188 Shizi Street, Suzhou 215006, China. E-mail address: wangzhong761@163.com or zqchen6@163.com

Cover title：Fluoxetine on Recovery after Acute Stroke

Tables 2; Figures 3, Word Count: 5626

Table S1: Inclusion, exclusion criteria, conclusion， data acquisition time and outcome assessments of the included studies

**Trails**	**Marquez-Romero et al 2020(NCT：01737541)**
Inclusion Criteria	1.Men and women ages ≥ 18 years<br>2.Individuals who meet one of the following criteria:<br>2.1. Patients who had an acute intracerebral hemorrhage within the past 10 days causing hemiparesis or hemiplegia<br>2.2. Fugl-Meyer motor scale (FMMS) scores ≤ 55<br>3.Written, informed consent for participation in the trial
Exclusion Criteria	1.Severe post-stroke disability (National Institutes of Health stroke scale [NIHSS] score >20)<br>2.Premorbid disability, evidenced by residual motor deficit from a previous stroke<br>3.Comprehension deficits or severe aphasia<br>4.Previous diagnosis of depression or one of the following:<br>Hospital anxiety and depression scale (HAD) score ≥11 points<br>Taking antidepressant drugs two weeks before inclusion<br>Taking neuroleptic drugs or benzodiazepines 2 weeks before inclusion<br>5.Other major diseases with life expectancy≤3 month
Efficacy Outcomes	1.FMMS total scores (TS) did not differ at baseline.<br>This was also the case for the upper (ULS) and lower limbs<br>(LLS) separate scores. Nevertheless, at day 90 the three scores were<br>statistically different between groups<br>2.At day 90, NIHSS scores were reduced a median of 3 points in the<br>treatment group vs 0 in the placebo group, nevertheless this difference<br>did not reach statistical significance (p = 0.131).<br>the proportion of patients within categories of the mRs≤2 as well as the medians<br>for Barthel Index scores at day 90 were statistically different between<br>groups.
Safety Outcomes	There were no serious adverse events during follow-up, all adverse events recorded were mild and no patient discontinued the study medication. The most common reported side effect was drowsiness.
Conclusion	There might still be a potential role for SSRI´s as an aid to motor recovery in ICH patients.
Data acquisition time	FMSS and NIHSS were measured at inclusion, at day 42 ± 7 days and at day 90.<br>BI and mRs were registered at day 90 ± 7 days

**Trails**	**Lundström, E et al 2020 (NCT02683213) EFFECTS**
Inclusion Criteria	Patients were eligible if they were aged 18 years or older with a clinical diagnosis of ischaemic or intracerebral haemorrhage in the previous 2–15 days; if brain imaging was compatible with intracerebral haemorrhage or ischaemic stroke; and the patient had at least one persisting focal neurological deficit severe enough (from the perspective of both the randomising physician and patient) to warrant treatment with fluoxetine for 6 months
Exclusion Criteria	Patients were excluded if they had a primary subarachnoid haemorrhage; were unlikely to be available for follow-up for the next 12 months;had a history of epileptic seizures; had previous drug overdose or attempted suicide; had an ongoing depression; were taking antidepressant medication, regardless of indication; were taking medications that could have a serious interaction with fluoxetine; had an allergy or contraindication to fluoxetine; had hepatic impairment (alanine aminotransferase more than 3 times the upper limit of normal) and renal impairment (creatinine >180 µmol/L); or were pregnant or breastfeeding.
Efficacy Outcomes	1.The primary outcome, which was centrally assessed, was functional status at 6 months (plus or minus 14 days) measured using the mRS.<br>2.The following secondary outcomes were centrally assessed at the trial coordinating centre at Danderyd Hospital at 6 months: survival and score in the assessed domains of the SIS version<br>3.The following secondary outcomes were assessed at face-to- face follow-up visits (not centrally assessed): NIHSS score; MoCA score; new diagnosis of depression; and adherence to trial medication(adherence was defined as taking the study medication 5–7 days per week, intermediate adherence was defined as taking the study medication 1–4 days per week or with some interruptions).
Safety Outcomes	These safety outcomes were new stroke (ischaemic or haemorrhagic), acute coronary events, upper gastro intestinal haemorrhage, new bone fractures, epileptic seizures, hyponatraemia (<130 mmol/L), and badly con trolled diabetes . Retrospectively, we also categorised patients with other serious bleeds and thrombotic events (pulmonary embolism, arterial embolism), which led to hospital admission. These were recorded at the local centres as serious adverse events.
Conclusion	the results from the EFFECTS trial show that oral fluoxetine 20 mg given once daily for 6 months after an acute stroke did not improve patients' functional Outcomes.
Data acquisition time	1MRS and SIS were measured by postal questionnaire or via interview over the telephone at 6 months after randomisation.<br>2.NIHSS and MoCA were measured at 3months and 6months face-to-face

Trails	Hankey, G. J et al 2020 (ACTRN12611000774921) AFFINITY
Inclusion Criteria	Eligible patients were adults (aged ≥18 years) with a clinical diagnosis of acute stroke within the previous 2–15 days, brain imaging consistent with ischaemic or haemorrhagic stroke, and a persisting neurological deficit that produced a mRS score of 1 or more.
Exclusion Criteria	1.they had any definite indication for fluoxetine,or contraindication to fluoxetine (eg, history of epilepsy, bipolar disorder, drug overdose, fluoxetine allergy, or recent medication that could interact with fluoxetine;<br>2.biochemical evidence of hepatic impairment [serumalanine aminotransferase concentration >120 U /L], renal impairment [creatinine concentration >180 μmol/L, estimated glomerular filtration rate <30 mL/min per 1·73 m²], or hyponatraemia[sodium concentration <125 mmol/L]);<br>3.unlikely to be available for follow-up during the subsequent 12 months<br>had another life-threatening illness that would make 12-month survival unlikely (eg, terminal malignancy);<br>4.if women were pregnant,breast-feeding, or of child-bearing age and not using contraception;<br>5.if patients were enrolled in another clinical trial of an investigational medicinal product or device
Efficacy Outcomes	1.months after randomisation.In Australia and New Zealand, the primary outcome was centrally assessed;in Vietnam, it was assessed by the site investigator.<br>2.Secondary outcomes at 6 months were survival, depression (change in PHQ-9 score from baseline and PHQ-9 score ≥15), cognition (TICSm score), communi cation, motor function, and overall health status (SIS score), fatigue (vitality subscale of the SF-36), health related quality of life (EQ-5D-5L), new diagnosis of depression requiring treatment with anti depressants, and trial medication adherence and cessation.
Safety Outcomes	These adverse events were any recurrent stroke (ischaemic or haemorrhagic), acute coronary syndromes, upper gastrointestinal bleeding requiring blood trans fusion or endoscopy, other major bleeding (subdural, extra dural, ocular, or lower gastrointestinal) requiring blood transfusion or procedural intervention, falls with injury, new bone fractures, epileptic seizures, symptomatic hypoglycaemia (blood glucose <3 mmol/L), symptomatic hyperglycaemia (blood glucose >22 mmol/L), new hypo natraemia (blood sodium <125 mmol/L), attempted suicide or self-harm, and death.
Conclusion	These results do not support the use of fluoxetine to improve functional outcome after stroke
Data acquisition time	MRS， PHQ-9， TICSm， SIS,SF-36,EQ-5D-5L were measured by the site investigator at 28, 90, and 180 days after randomisation

**Trails**	**Dennis, M et al 2019 (ISRCTN83290762) FOCUS**
Inclusion Criteria	1.aged 18 years or older<br>2.had a clinical diagnosis of acute stroke with brain imaging compatible with intracerebral haemorrhage or ischaemic stroke (including a normal brain scan)<br>3.were randomly assigned between 2 days and 15 days after stroke onset<br>4.had a persisting focal neurological deficit at the time of randomisation that was severe enough to warrant 6 months of treatment
Exclusion Criteria	1.had subarachnoid Haemorrhage except where secondary to a primary intracerebral haemorrhage;<br>2.were unlikely to be available for follow-up for the following 12 months<br>3.were unable to speak English and had no close family member available to help with follow-up;<br>4.had another life-threatening illness that would make 12-month survival unlikely;<br>5.had a history of epileptic seizures; they had a history of allergy to fluoxetine<br>6.had contraindications to fluoxetine, including hepatic impairment or renal impairment<br>7.were pregnant or breastfeeding, or women of childbearing age not taking contraception<br>8.had a previous drug overdose or attempted suicide<br>9.were already enrolled into a controlled trial of an investigational medicinal product;<br>10.had current or recent (within the last month) depression treated with an SSRI<br>11.were taking or had, in the past 5 weeks, taken medications that have a potentially serious interaction with fluoxetine.
Efficacy Outcomes	1.The primary outcome was functional status, measured with the mRS, at the 6-month follow-up. We used the simplified mRS questionnaire (smRSq) delivered by post.<br>2.There were no significant differences in any other secondary outcomes at 6 months, including any of the nine domains of the SIS, the Vitality subscale of SF36, and EQ5D-5L (table 2) or other recorded adverse reactions
Safety Outcomes	adverse events at 6 months
Conclusion	These results do not support the routine use of fluoxetine either for the prevention of post-stroke depression or to promote recovery of function.
Data acquisition time	National coordinating centre staff followed up patients at 6 months and 12 months to measure the primary and secondary outcomes.<br>Data on adverse events and medications were also collected from patients' general practitioners at 6 months and 12 months.

**Trails**	Chollet, F et al2011 (NCT:00657163) FLAME
Inclusion Criteria	1.had an acute ischaemic stroke within the past 5–10 days that caused hemiparesis or hemiplegia<br>2.were prospectively enrolled from nine stroke units in France.<br>3.aged between 18 years and 85 years<br>4.had Fugl-Meyer motor scale (FMMS) scores of 55 or less at baseline were eligible for inclusion.
Exclusion Criteria	1.had severe post-stroke disability (National Institutes of Health stroke scale [NIHSS] score >20), substantial premorbid disability, or a preexisting deficit that could interfere with assessments<br>2.clinically diagnosed with depression or Montgomery Åsberg depression rating scale (MADRS) score of more than 19<br>3.taking antidepressant drugs, monoamine oxidase inhibitors, neuroleptic drugs, or benzodiazepines during the month<br>4.before inclusion or due to underg carotid endarterectomy.<br>5.Other exclusion criteria included pregnancy and other major diseases that would prevent follow-up
Efficacy Outcomes	1.The primary outcome was the mean change in FMMS score between inclusion (day 0) and day 90<br>2.Secondary endpoints were NIHSS,MRS,and MADRS
Safety Outcomes	adverse events and deaths within 3 months after randomisation
Conclusion	We noted a positive eff ect on motor recovery in patients with acute ischaemic stroke who were treated with fluoxetine for 3 months
Data acquisition time	FMMS,NIHSS,MRS and MADRS were measured at baseline, day 30, and day 90.

Trails	Bonin Pinto, C et al 2019 (NCT:02208466)
Inclusion Criteria	adults with hemiparesis/hemiplegia due to ischemic stroke within the previous 2 years, upper extremity Fugl-Meyer (FMA) motor score >11 and <56, and prestroke disability score <3 on Modified Rankin Scale (mRS).
Exclusion Criteria	unable to understand instructions, TMS contraindications,concurrent medical condition likely to worsen functional status within 6 months, score of 24 or higher on Hamilton Depression Rating Scale (HDRS), joint or paretic extremity pain, pregnancy, recent intake of SSRI or any medication likely to interact with SSRIs (5-week washout period was required before enrollment in either case).
Efficacy Outcomes	1.The primary outcomes were motor function scales: JTHF and FMA upper extremity changes from day 0 to day 90.<br>2.Secondary Outcomes. Modified Ashworth Scale (MAS), Beck's Depression Inventory (BDI), Mini-Mental State Examination (MMSE), medication diary,
Safety Outcomes	antidepressant side effect, and rTMS side effects questionnaires
Conclusion	Combined fluoxetine and rTMS treatment leads to better motor function in stroke than fluoxetine alone and placebo. Moreover, fluoxetine leads to smaller improvements than placebo
Data acquisition time	All outcomes were assessed on day 0 (baseline visit), day 30, and day 90 .

**Trails**	**Asadollahi, M et al 2018 (IRCT20141116019971N3)**
Inclusion Criteria	1.over 18 years of age,<br>2.suffering from hemiparesis or hemiplegia as a result of a first-time acute ischemic stroke within the past 24 hours,<br>3.an initial Fugl-Meyer Motor Scale score10 of under 55.
Exclusion Criteria	1.a National Institutes of Health Stroke Scale score11 of less than five;<br>2.prior disabilities including aphasia, cognitive disorders and motor disorders<br>3.due to stroke, or any other neurodegenerative disease<br>4.pregnancy or breastfeeding currently taking antidepressants<br>5.contraindications of therapy, including renal insufficiency (glomerular filtration rate <30 mL/min), abnormal liver function tests, hyponatremia, and a long QT interval on an electrocardiogram;<br>6.any significant adverse effects (agitation, hypertension, or other signs of serotonin syndrome) after initiation of treatment.
Efficacy Outcomes	after 90 days, compared with the placebo group, the mean Fugl-Meyer Motor Scale score showed significant increases in the fluoxetine and citalopram groups (P = 0.001).
Safety Outcomes	The most frequently reported side-effect of the treatments was increased appetite.
Conclusion	There was no significant difference between citalopram and fluoxetine in facilitating post-stroke motor recovery in ischemic stroke patients. However, compared with a placebo, both drugs improved post-stroke motor function.
Data acquisition time	Fugl-Meyer Motor Scale score at the enrollment, and at days 30, 60, and 90 after the intervention.<br>They also recorded any adverse events during the follow-up.

Trails	He, Y. T et al 2016 (ChiCTR-TRC-12002078)
Inclusion Criteria	1.adherence to the diagnostic criteria of ischemic stroke as defined by the World Health Organization,<br>2.first onset of stroke within 1 week,<br>3.age 18-80 years,<br>4.symptoms of defective nervous system and National Institutes of Health Stroke Scale (NIHSS) score higher than2,<br>5.informed consent of patients or their legal relatives.
Exclusion Criteria	1.hemorrhagic stroke;<br>2.coma;<br>3.obvious symptoms of defective nervous system before stroke onset;<br>4.existing self-injury or suicidal tendency , and urgent need of antidepressants;<br>5.use of antidepressants within 3 months before the onset or benzodiazepines within 2 weeks before the onset;<br>6.serious heart diseases, respiratory failure, malignant tumor;<br>7.active peptic ulcer or other gastrointestinal diseases, which may affect drug absorption;<br>8.abnormal liver enzymes or creatinine;<br>9.pregnancy or lactation;<br>10.severe mental disorders,such as schizophrenia;<br>11.allergic constitution;<br>12.participation in other clinical studies within 3 months before the study;<br>13.scheduled endovascular intervention
Efficacy Outcomes	1.There were no significant differences in the mean NIHSS scores at baseline, day 15, and day 90, and in the mean BI scores at baseline between the 2 groups. However, the mean NIHSS score on day 180 in the treatment group was significantly lower than that in the control group (P = .009).<br>2.Meanwhile, the mean BI scores on days 90 and 180 in the treatment group were significantly higher than those in the control group, respectively (P= .026, P= .011)
Safety Outcomes	During the follow-up, 3 patients in the control group and 4 patients in the treatment group developed hemorrhagic infarction, which was not significantly different between the groups (P = .690).<br>The mortality rate in the 2 groups was not significantlydifferent
Conclusion	Treatment with fluoxetine for 90 days after ischemic stroke can improve the long-term neural functional outcomes.
Data acquisition time	NIHSS score at enrollment, day 15, 90, 180, and BI score at enrollment, day 90 and 180.

Trails	Mikami, K et al 2011
Inclusion Criteria	1.had a strok in the previous 6 months,e were screened for participation in a study of antidepressant therapy .<br>2.The protocols were approved by the institutional review boards and written informed consent was obtained from each subject.<br>3.In addition,we required that the subject's immediate family and treating physician also agreed to the subject's participation.
Exclusion Criteria	1.any other significant medical illness that would threaten the patient's life or recovery from stroke;<br>2.severe comprehension deficit that precluded a verbal interview (defined as making an error on part 1 of the Token Test);<br>3.prior history of other brain diseases,with the exception of prior stroke.
Efficacy Outcomes	During the 1-year follow-up period, and after adjusting for critical confounders including age, intensity of rehabilitation therapy, baseline stroke severity,and baseline Hamilton Depression Rating Scale, patients who received fluoxetine or nortriptyline had significantly greater improvement in modified Rankin Scale scores compared to patients who received placebo($t[156] = -3.17$, $p = 0.002$)
Safety Outcomes	one subject had severe anxiety, two had severe gastrointestinal symptoms, and one had insomnia
Conclusion	the administration of either nortriptyline and fluoxetine for 3 months significantly reduced disability from stroke over 1 year compared with placebo, even after controlling for age, total hours of physical rehabilitation, baseline severity of stroke (using the NIHSS), and baseline HDRS score
Data acquisition time	time points (0, 3, 6, 9,12-months; treated as a continuous measure)

Trails	Guo, Y et al 2016 (ChiCTR-IPR-15007658)
Inclusion Criteria	1.Adherence to the diagnostic criteria of ischemic stroke as defined by World Health Organization (WHO) (Kunitz et al., 1984).<br>2.18–80 years of age.<br>3.First onset of stroke within 1 week.<br>4.Symptoms of defective nervous system and NIHSS score >2.<br>5.Informed consent from patients or from their legal relatives
Exclusion Criteria	1.Coma.<br>2hemorrhagic stroke.<br>3.Symptoms of defective nervous system before the onset of stroke.<br>4.Use of antidepressants within 3 months or benzodiazepines within 2 weeks before the onset of stroke.<br>5.Self-injurious or having suicidal tendency, and urgency of antidepressants.<br>6.Serious heart diseases, respiratory failure, malignant<br>tumors.<br>7.Abnormal liver enzymes or creatinine levels.<br>8.Active peptic ulcer or other gastrointestinal diseases, which may affect the absorption of drugs.<br>9.Allergic constitution.<br>10.Severe mental disorders, such as schizophrenia.<br>11.Pregnant or lactating women.<br>12.Participated in other clinical studies within 3 months before the onset of the study.<br>13.Planned endovascular treatment.<br>14.Patients or their legal relatives who refused to participate in the study.
Efficacy Outcomes	1.The mean NIHSS score at day 90 was significantly lower in group A than group C (P= 0.005), while at day 180, the mean score in group A was significantly lower than groups B and C (P= 0.035, P= 0.000), respectively.<br>2.The mean BI score at day 90 was significantly higher in group A than group C (P= 0.001), while at day 180, the mean score in group A was significantly higher than groups B and C (P= 0.036, P= 0.000), respectively.
Safety Outcomes	Intracranial hemorrhage was observed in one patient in group A,2 patients in both group B and group C, respectively;however, occupied hematoma was not observed in any of the patients. The incidence of intracranial hemorrhage in the three groups were not significantly different .<br>The mortality rates in the three groups were not significantly different.
Conclusion	In patients with ischemic stroke, early administration of fluoxetine may improve the neurological functional prognosis.
Data acquisition time	The NIHSS score at days 15, 90, and 180 after enrollment<br>The BI score at days 90 and 180 after enrollment

Trails	Pariente, J et al 2001
Inclusion Criteria	1.Age 40 to 80 years<br>2.Pure motor hemiparesia secondary to an acute lacunar infarct, localized on the pyramidal tract<br>3.Patient able to perform a flexion–extension task with fingers of the affected arm<br>4.Confirmation and localization of stroke by CT scan or MRI<br>5.Informed consent obtained
Exclusion Criteria	1.Patients suffering from other major disease(s)<br>2.Allergy to SSRI or other related drugs<br>3.Stroke associated with clinical sensory loss or cortical symptoms such as aphasia or neglect.<br>4.Patients with contraindication to MRI<br>5.Alcoholism or drug addiction
Efficacy Outcomes	Finger tapping and clinical scales presented only as graphs. fMRI activation location before fluoxetine or placebo intake and 5 hours later (a few minutes before the fMRI examination)<br>The nine peg hole test, dynamometer, and finger tapping.
Safety Outcomes	N/E
Conclusion	We have shown a pharmacological modulation of cerebral motor activation correlated to an improvement of motor performance in 8 patients with a single lacunar infarct. Further studies are required to investigate the effect of a single dose of fluoxetine on patients with stroke, including the MI cortex, and also to investigate the effect of chronic administration of fluoxetine on motor function and on cerebral activation.
Data acquisition time	Each subject underwent two fMRI examinations , the first 14 days (range, 7–23 days) after stroke onset, and the second 7 days later. These two examinations were processed 5 hours after administration of fluoxetine (at peak plasma concentration) or placebo, given at 8 AM.

Trails	Robinson, R. G et al 2000
Inclusion Criteria	Inclusion criteria included acute stroke within 6 months of the onset of the study and age 18–85
Exclusion Criteria	1.any other significant medical illness that would threaten the patient's life or recovery from stroke,<br>2.severe comprehension deficit that precluded a verbal interview (defined as failing part 1 of the Token Test),<br>3.prior history of head injury,<br>4.prior history of other brain disease with the exception of prior stroke(N=103 were excluded).
Efficacy Outcomes	HDRS, mRS, FIM, MMSE, JHFI,<br>death, AEs
Safety Outcomes	In the nondepressed fluoxetine group, four patients dropped out—two during the first 3 weeks, and two between weeks 4 and 6. (One complained of gastrointestinal symptoms, two deteriorated medically, and one refused treatment.)<br>One of the nondepressed placebo patients dropped out between weeks 7 and 9. (The patient developed a rash.)
Conclusion	Neither depressed or nondepressed patients treated with nortriptyline or fluoxetine showed significantly greater improvement in their stroke-associated impairments than patients treated with placebo.
Data acquisition time	The follow-up visits were conducted in the treating hospital or , most often, in the patient's home or long-term-care facility each 3-week

Trails	Dam, M et al 1996
Inclusion Criteria	Subjects unable to walk 1 to 6 months after a CT-proven hemispheric ischemic stroke in the territory of the middle cerebral artery were considered eligible for the study.
Exclusion Criteria	Patients with a history of major affective disorders or Alcohol abuse or a history and/or clinical evidence of severe heart,lung,kidney,or liver diseases or mental deterioration were excluded.
Efficacy Outcomes	HDRS, HSS (total, gait and motor scores), BI
Safety Outcomes	death, AEs
Conclusion	Fluoxetine and maprotiline may differently influence functional outcome after ischemic brain injury
Data acquisition time	HDRS,HSS and BI scores were determined at baseline and 90 days

Figure S1. Sensitivity analysis of the mRS (0-2)

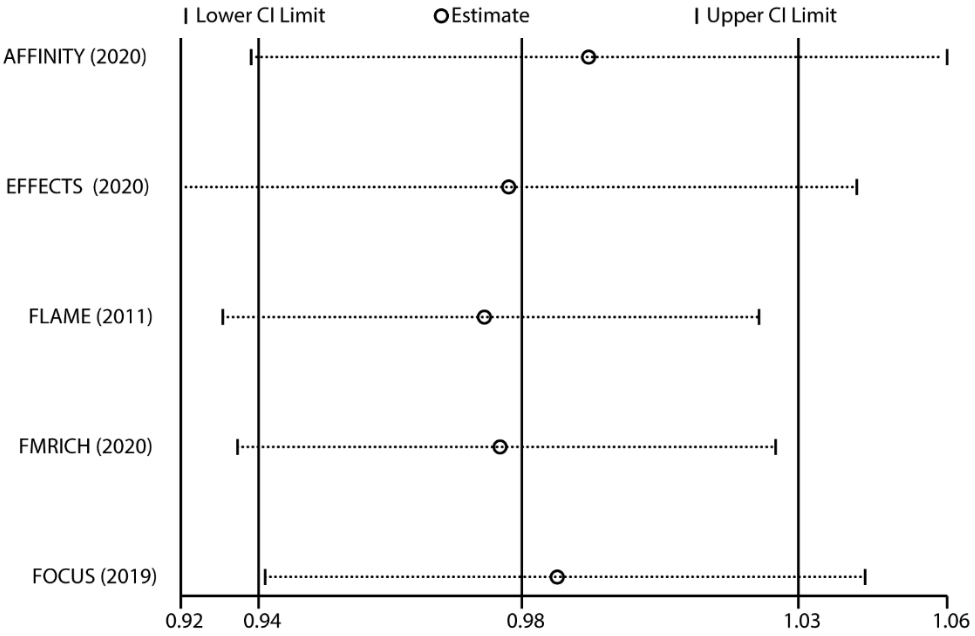

Figure S2. BI without He 2016

	fluoxetine			placebo				Std. Mean Difference
Study or Subgroup	Mean	SD	Total	Mean	SD	Total	Weight	IV, Fixed, 95% CI
Dam 1996	23.5	12.2	16	19.1	18.27	16	12.0%	0.28 [-0.42, 0.97]
Guo 2016	31.68	17.18	177	22.67	22.01	90	88.0%	0.47 [0.22, 0.73]
He 2016	32.12	17.04	177	27.65	19.16	170	0.0%	0.25 [0.04, 0.46]
**Total (95% CI)**			**193**			**106**	**100.0%**	**0.45 [0.21, 0.69]**
Heterogeneity: Chi² = 0.27, df = 1 (P = 0.60); I² = 0%								
Test for overall effect: Z = 3.66 (P = 0.0002)								

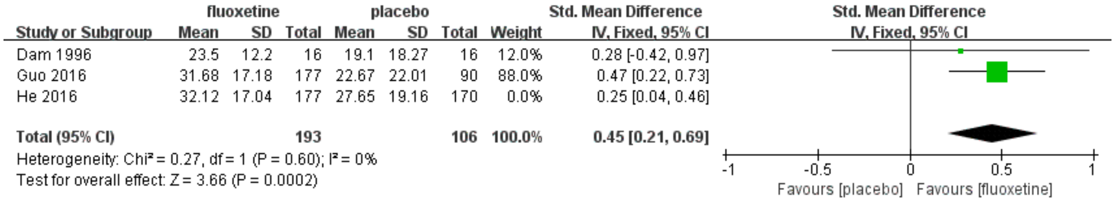

Figure S3. NIHSS without He 2016

	fluoxetine			placebo				Std. Mean Difference
Study or Subgroup	Mean	SD	Total	Mean	SD	Total	Weight	IV, Fixed, 95% CI
EFFECTS 2020	-2	2.56	678	-2	2.56	681	78.3%	0.00 [-0.11, 0.11]
FLAME 2011	-7	3.8	57	-6.2	4.35	55	6.4%	-0.19 [-0.57, 0.18]
FMRICH 2020	-3.5	3.23	14	-2	4.63	16	1.7%	-0.36 [-1.09, 0.36]
Guo 2016	-5.47	2.83	177	-4.66	3.22	90	13.6%	-0.27 [-0.53, -0.02]
He 2016	-5.54	2.84	177	-4.96	3.27	170	0.0%	-0.19 [-0.40, 0.02]
**Total (95% CI)**			**926**			**842**	**100.0%**	**-0.06 [-0.15, 0.04]**
Heterogeneity: Chi² = 5.05, df = 3 (P = 0.17); I² = 41%								
Test for overall effect: Z = 1.16 (P = 0.25)								

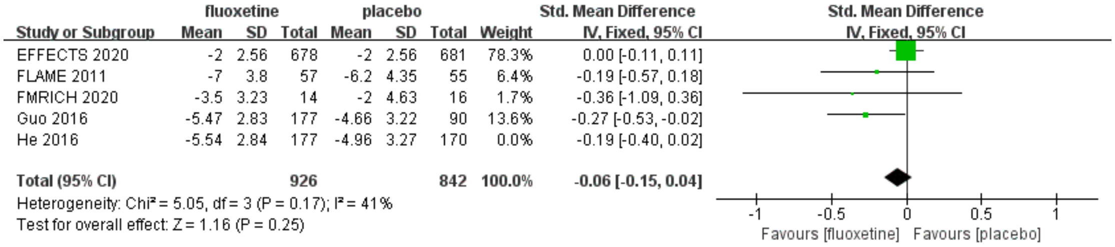

Supplement: Supplementary file 1 [file Data_Sheet_1.PDF]
